# Epidemiology and antimicrobial resistance of toxin‐producing *Klebsiella oxytoca* clinical isolates from children admitted to the oncology chemotherapy center in Mofid Children's Hospital in Tehran, Iran: A cross‐sectional study

**DOI:** 10.1002/hsr2.2275

**Published:** 2024-07-31

**Authors:** Nasim Sabzivand, Shiva Nazari, Fariba Shirvani, Leila Azimi, Siavash Salmanzadeh Ahrabi, Maedeh Mohammadi Estiri

**Affiliations:** ^1^ Department of Microbiology, Faculty of Biological Sciences Alzahra University Tehran Iran; ^2^ Pediatric Congenital Hematologic Disorders Research Center, Mofid Children Hospital Shahid Beheshti University of Medical Sciences Tehran Iran; ^3^ Pediatric Infections Research Center, Research Institute for Children's Health Shahid Beheshti University of Medical Sciences Tehran Iran; ^4^ Medicine School Shahid Beheshti University of Medical Sciences Tehran Iran

**Keywords:** antimicrobial resistance, epidemiology, Iran, *Klebsiella oxytoca*, toxin

## Abstract

**Background and Aims:**

*Klebsiella oxytoca* (*K. oxytoca*) is the second bacterial cause of nosocomial infections in the general population after *K. pneumoniae*. This study surveyed the frequency of cytotoxin‐producing strains of *K. oxytoca* and their antibiotic susceptibility profile in a cohort of children admitted to a referral hospital with different malignancies.

**Methods:**

The Stool samples of children admitted to the Cancer Chemotherapy Unit of the Mofid Children's Hospital, Tehran, Iran were analyzed using conventional biochemical tests and polymerase chain reaction targeting the *pehX* gene to identify *K. oxytoca*. The antibiotic susceptibility profile of isolated *K. oxytoca* against commonly prescribed antibiotics used in treating infection at the facility was determined using the Kirby–Bauer disk diffusion technique. Also, the prevalence of genes encoding toxins among *K. oxytoca* was identified by PCR assay.

**Results:**

The Stool samples of 280 participants were taken for the study of which 38 samples [(55.3% (21/38) 42 males and 44.7% (17/38) females)] tested positive for various *Klebsiella* spp. Out of this, *K. oxytoca* was identified in 2.5% (7/280) stools using cultures and conventional biochemical tests. Also, the stools of 2.9% (8/280) of the participants tested positive for *K. oxytoca* using PCR assay. Using PCR, (2/7) of the *K. oxytoca* isolates tested positive for the *npsA* and *npsB* genes and were identified as toxigenic *K. oxytoca* strains.

**Conclusion:**

The prevalence of toxin‐producing *K. oxytoca* strains in stool samples of children diagnosed with cancer in Iran is relatively low. Most of the *K. oxytoca* isolates were susceptible to tested antibiotics. Globally, active surveillance of toxigenic *K. oxytoca* strains in patients with different malignancies or immunocompromised patients is recommended in healthcare settings.

## 1 INTRODUCTION

*Klebsiella* spp. are gram‐negative, facultatively anaerobic, oxidase‐negative, non‐spore‐forming, and rod‐shaped bacteria belonging to the order Enterobacterales.^1^ *Klebsiella* spp. consists of different species such as *Klebsiella pneumoniae* (*K. pneumoniae*), *K. oxytoca*, *K. aerogenes*, *K. granulomatis*, *K. michiganensis*, *K. quasipneumoniae*, *K. grimontii*, and *K. variicola*.^2^, ^3^ *Klebsiella* species are opportunistic pathogens in hospital and community‐acquired infections.^4^ *K. oxytoca* is a part of the human gut microflora; yet, this bacterium is regarded as the second bacterial cause of nosocomial and community‐acquired human illnesses after *K. pneumoniae*.^5^, ^6^ *K. oxytoca* is implicated in the pathophysiology of numerous infections in people with compromised immunity, children, and newborns.^7^, ^8^, ^9^ In healthcare facilities, *K. oxytoca* causes between 13% and 24% of nosocomial bacteremia.^2^

In recent years, multidrug resistant (MDR) *K. oxytoca* has been increasingly isolated from healthcare settings.^2^ *K. oxytoca* multidrug resistance is achieved through either the expression of an efflux pump or the enzymatic degradation of antibiotics. In addition, *K. oxytoca* applies different antibiotic resistance mechanisms such as enzymatic degradation of antibiotics and efficient removal of antibiotics through efflux pumps as part of its multidrug resistance mechanism.^10^

The pathogenicity of *K. oxytoca* is associated with the production of tilivallines, a cytotoxin that damages the intestinal epithelium.^11^, ^12^ Tilivalline is a pentacyclic pyrrolobenzodiazepine metabolite of the *K. oxytoca* ^2^ and the synthesis of this cytotoxin occurs through a bimodular nonribosomal peptide synthetase (NRPS) pathway that has three proteins: NpsA, ThdA, and NpsB.^13^, ^14^ Biosynthesis, mechanism of action of the Enterotoxin Tilivalline produced by the *K. oxytoca* has been explained in a study performed by Alexander et al. in 2020.^13^

Despite clinical relevance of *K. oxytoca* and the increasing concerns about cytotoxin‐producing strains, little is known about the epidemiology and antibiotic resistance profile of *K. oxytoca* within healthcare institutions in Iran. Therefore, the current study surveyed the frequency of cytotoxin‐producing strains of *K. oxytoca* and their antibiotic susceptibility profile in a cohort of children admitted with different malignancies into a referral hospital in Tehran, Iran.

## 2 MATERIALS AND METHODS

### 2.1 Ethics statement

The Pediatric Infections Research Center Ethics Committee, Research Institute for Children's Health, Shahid Beheshti University of Medical Sciences, Tehran, Iran, approved the current study (Ref no: 04365). The purposes of the study was explained to the parents/caregivers of the study participants after which a signed written informed consent was obtained from the parents/caregivers.

### 2.2 Study design and sample collection

This facility‐based cross‐sectional study was conducted from March 1, 2021, to May 31, 2022, among hospitalized children at the Oncology Chemotherapy Unit, Mofid Children's Hospital, Tehran, Iran. Mofid Children's Hospital is a medical, educational and therapeutic center in Tehran, Iran. This center has been introduced as a “Child‐Friendly Hospital” by the UNICEF and with 420 active beds admits a significant population of sick children from different parts of the country as well as neighboring countries (http://en.mch.sbmu.ac.ir/).

Stool containers were labeled with the participant's name, date, and time and was given to the parent/caregiver to collect stool samples from the study participants. The collected stool samples were subsequently sent to the Shahid Beheshti University of Medical Sciences' Pediatric Infections Research Center, Iran, part of the Research Institute for Children's Health, for laboratory analysis. The stool sampling process was supervised by Nasim Sabzivand and Fariba Shirvani. All demographic data of participants was recorded through the design of a data collection form.

### 2.3 Study participants

The study participants were children with different malignancies and admitted to the oncology chemotherapy unit at Mofid Children's Hospital, Tehran, Iran. All study participants had a history of hospitalization 6 months before sampling. The list of different malignancies and underlying diseases among study participants is shown in Table 2. Children who have incomplete medical records and children who did not sign written informed consent were excluded from the study.

### 2.4 Phenotypic identification of *Klebsiella* spp

A sterile cotton wool swab stick was used to collect about 1 g of each stool sample and dissolved in 3 mL physiological saline.^15^ This was thoroughly mixed to achieve consistency and streaked on Eosin methylene blue and MacConkey agar (Merck) and incubated overnight at 37°C for 18–24 h.

The plates were checked for the presence of a colony, and the observation of a colony on the medium was considered positive bacterial growth. The bacterial colonies harvested using a sterile loop and Gram staining was performed on all bacterial colonies. Briefly, a thin layer of bacteria was smeared on a slide and air‐dried. In the Gram staining assay, crystal violet stain was applied on the prepared slide for 60 s, after which iodine solution was applied for 60 s. Ethanol and acetone as a decolourizer were added for 5 s, and basic fuchsin solution was added for 45 s.^16^ The presumptive identification of *Klebsiella* spp. was done using conventional biochemical tests including catalase and oxidase tests, Indole, Methyl red, Voges proskauer, and Citrate tests (IMVIC), growth on Triple Sugar Iron (TSI) agar, SH2 production, motility test, urease test, lysine decarboxylase, and ortho‐nitrophenyl‐β‐galactopyranoside (ONPG). Trypticase soy broth (TSB) with 20% glycerol was used as a freezing medium for the storage of *Klebsiella* spp.^17^

### 2.5 Molecular detection of the *pheX* gene

For this purpose, 100 mg of stool sample was dissolved in 1 mL of physiological serum and thoroughly mixed to achieve consistency, after which 100 µL the mixture was aliquoted into a 1.5 mL microtube and 100 µL of protease storage buffer added into the suspension and incubated at 55 C for 30 min.^18^ Following the manufacturer's instructions, total genomic DNA was isolated using a DNA extraction kit (Sinaclon Co.). Until usage, all extracted DNA was kept at −80°C. The conventional polymerase chain reaction (PCR) approach was used for the definitive identification of *K. oxytoca*. PCR assay targeted the polygalacturonase (*pehX*) gene with primers: Forward: 5‐GATACGGAGTATGCCTTTACGGTG‐3 and Reverse: 5‐ TAGCCTTTATCAAGCGGATACTGG‐3 (344 base pairs). The PCR reaction was performed at a final volume of 25 μL with 11 µL of 2x Master mix (Amplicon, Sinaclon Co.), 0.5 μL of 10 pmol of each forward and reverse primer, 5 µL of extracted DNA, and 8 µL of sterile distilled water. PCR(s) was performed on a thermocycler (Applied Biosystems) under the following conditions: initial denaturation step: 1 cycle of 95°C for 2 min, denaturation step: 30 cycles of 94°C for 20 s, annealing step: 30 cycles of 59°C for 20 s, extension step: 30 cycles of 72°C for 30 s, and final extension step: 1 cycle of 72°C for 10 min. Finally, 1% agarose gel was prepared and stained with a DNA‐safe stain (Sinaclon Co.). All PCR products were screened on a agarose gel and photographed under UV transilluminator (Life Technologies).

### 2.6 Antimicrobial susceptibility testing

The susceptibility of *K. oxytoca* strains to ampicillin (10 μg), imipenem (10 μg), meropenem (10 μg), ciprofloxacin (5 μg), ampicillin‐sulbactam 20 μg, trimethoprim‐sulfamethoxazole (25 μg), amikacin (30 μg), ceftriaxone (20 μg), cefazolin (30 μg), levofloxacin (5 μg), and cefixime (5 μg) (MAST) was determined by Kirby–Bauer disk diffusion method on a Mueller Hinton agar (Merck). The choice of the antibiotic and interpretation of the results was performed according to the Clinical and Laboratory Standards Institute (CLSI, 2020). The results were interpreted as either susceptible, intermediate, or resistant. *Escherichia coli* ATCC 25922 was used as the quality control strain.

### 2.7 Detection of toxin‐producing *K. oxytoca*

The prevalence of toxin‐producing *K. oxytoca* strains among the study participants was identified using conventional PCR assays with specific primers targeting the *npsA* and *npsB* genes which are specific to *K. oxytoca*. Table 1 shows the primer sequences used in the PCR assay. The final volume of the PCR reaction was 25 µL containing 12 µL of 2x Master Mix (Amplicon, Sinaclon Co.), 0.5 µL of forward primer (10 pmol), 0.5 µL of reverse primer (10 pmol), 4 µL of template DNA, and 8 µL of sterile distilled water. PCR conditions were set based on a previously published study by Cosic et al.^19^ PCR products were stained with a DNA‐safe stain (Sinaclon Co.) and visualized on a 1.5% gene agarose.

**Table 1:** Primer sequences used in the identification of pathogenic genes sequence in isolated *K. oxytoca*.

Genes		Primer sequences	Product size (bp)
*npsA*	F	5′‐TCGCAACGTTTTCCGGACAGGGTTG‐3′	299
	R	5′‐CACGCTTGTTACATCATCGCTA‐3′	
*npsB*	F	5′‐AATGTGGTGGCTGGATAATACGCTG‐3′	260
	R	5′‐AGCTAATGATAAACGGCTAGC‐3′	

### 2.8 Statistical analysis

All data were entered into the statistical package SPSS v.23.0 (SPSS Inc.) and reported using descriptive statistic tests.

## 3 RESULTS

### 3.1 Participants' characterization and demographic data

Two hundred and eighty participants (61.4% (172/280) males and 38.6% (108/280) female) from which stool samples were taken took part in the study. The mean age of the participants was 8.23 ± 1.15 years with 154 (55%), 83 (29.6%), and 43 (15.4%) participants aged 1–5, 6–10, and 11–15 years, respectively. The most common malignancies identified among the study participants were acute lymphocytic leukemia (ALL) 19.6% (55/280), acute myelogenous leukemia (AML) 14% (148 39/280), neuroblastoma 11.5% (32/280), Wilms tumor 9.6% (27/280), and Burkitt lymphoma 7.1% (20/280). The mean duration of hospitalization was 3.5 ± 1.5 days (Table 2).

**Table 2:** Characterization and demographic data of study participants.

Characterization and demographic data				Frequency (%)
Age (year)	1–5	Mean ± SD: 8.23 ± 1.15		154 (55%)
	6–10			83 (29.6%)
	11–15			43 (15.4%)
Gender	Male			172 (61.4%)
	Female			108 (38.6%)
Underlying disease	Wilms tumor			27 (9.6%)
	Burkitt lymphoma			20 (7.2%)
	Brain tumor			9 (3.2%)
	Non‐Hodgkin lymphoma			3 (1%)
	Neuroblastoma			32 (11.4%)
	Hepatoblastoma			7 (2.5%)
	Schwannomas			4 (1.4%)
	Dysgerminoma			2 (0.7%)
	ALL			54 (19.3%)
	SCT			3 (1%)
	AML			39 (13.9%)
	PNET			2 (0.7%)
	HLH			1 (0.4%)
	LCH			14 (5%)
	Ewing sarcoma			6 (2.1%)
	PNH			2 (0.7%)
	Pelvic tumor			4 (1.4%)
	ITP			12 (4.3%)
	T‐cell lymphoma			5 (1.8%)
	Abdominal mass			6 (2.1%)
	Hodgkin lymphoma			3 (1%)
	Synovial sarcoma			10 (3.6%)
	CGD			5 (1.8%)
	Glanzmann thrombasthenia			4 (1.4%)
Duration of hospitalization (Day)	0–3		Mean ± SD: 3.5 ± 1.5	156 (55.7%)
	4–7			81 (28.9%)
	8–11			38 (13.6%)
	12–15			5 (1.8%)

Abbreviations: ALL, acute lymphoblastic leukemia; AML, acute myeloid leukemia; CGD, chronic granulomatous disease; HLH, hemophagocytic lymphohistiocytosis; ITP, immune thrombocytopenic purpura; LCH, Langerhans cell histiocytosis; PNET, primitive neuroectodermal tumor; PNH, paroxysmal nocturnal hemoglobinuria; SCT, sickle cell trait.

### 3.2 Prevalence of *K. oxytoca* strains

**Figure 1:**
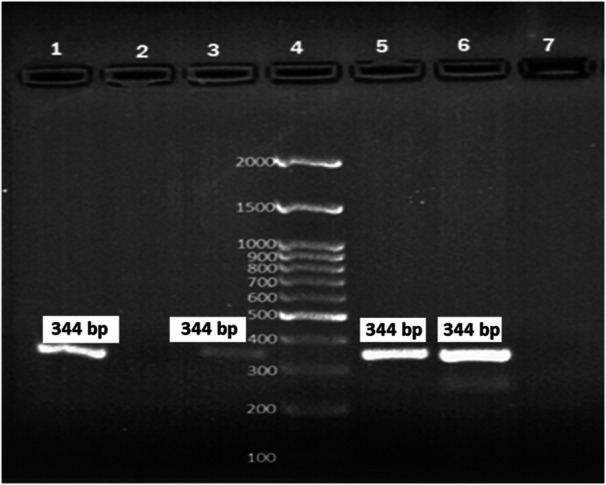
PCR‐based identification of the *pehX* gene. 4: DNA Ladder 100 bp, 1, 3, 5, and 6: Positive sample; 7: Negative control.

Using conventional biochemical tests, *Klebsiella* spp. was identified in the stool samples of 38 participants, of which 55.3% (21/38) and 44.7% (17/38) were males and females, respectively. The frequency of the *Klebsiella* species was as follows: *K. pneumoniae* (63.2%; 24/38), *K. oxytoca* (21%; 8/38), and other species of *Klebsiella* (15.8%; 6/38). PCR assay using the *pehX* gene confirmed 87.5% (7/8) of isolates as the *K. oxytoca* (Figure 1).

In total, *K. oxytoca* was detected in 2.5% (7/280) study participants using stool culture and conventional biochemical tests. In contrast, out of 280 stool samples, 2.9% (8/280) were positive for *K. oxytoca* using PCR (*pehX*) assay. The clinical and demographic data of the 8 participants who tested positive for *K. oxytoca* using PCR is shown in Table 3.

**Table 3:** The clinical and demographic data of eight participants who were positive for *K. oxytoca*.

Patients	Sex	Age (years)	Duration of hospitalization	Underlying disease	Diarrhea	Antibiotic usage history	Type of antibiotics (Duration)	Neutropenia	Neutrophil count
1	Female	4	9 days	Hepatoblastoma	No	Yes	Ceftazidime (6 days) Vancomycin (6 days) Amikacin (3 days) Tazocin (3 days)	Yes	400
2	Male	6	5 days	ALL	No	No	–	No	2300
3	Male	3	5 days	Wilms tumor	No	Yes	Ceftazidime (2 days) Meropenem (4 days)	No	2500
4	Male	12	2 days	Brain tumor	No	No	–	No	2300
5	Female	4	8 days	Wilms tumor	No	Yes	Meropenem (4 days)	No	1900
6	Male	9	6 days	Brain tumor	No	No	–	No	3000
7	Male	12	7 days	Brain tumor	No	No	–	No	2500
8	Male	12	42 days	Glanzmann thrombasthenia	No	No	–	No	2600

### 3.3 Antibiotic susceptibility testing and toxin profiling

Presented in Table 4 is the antibiotic susceptibility profile of isolated *K. oxytoca* strains against tested antibiotics. Most of the *K. oxytoca* strains were resistant to ampicillin (7/8; 87.5%) and cefazolin (5/8; 62.5%), respectively. In contrast, isolated *K. oxytoca* strains showed the highest levels of susceptibility (1/8; 12.5%) to imipenem, meropenem, ciprofloxacin, amikacin, and levofloxacin.

**Table 4:** Antibiotic susceptibility profile of eight *K. oxytoca* strains.

Antibiotics	Resistance	Intermediate	Susceptible
Ampicillin	7 (87.5%)	–	1 (12.5%)
Imipenem	1 (12.5%)	–	7 (87.5%)
Meropenem	1 (12.5%)	–	7 (87.5%)
Ciprofloxacin	1 (12.5%)	–	7 (87.5%)
Ampicillin‐sulbactam	2 (25.0%)	3 (37.5%)	3 (37.5%)
Trimethoprim‐sulfamethoxazole	4 (50.0%)	–	4 (50.0%)
Amikacin	1 (12.5%)	–	7 (87.5%)
Ceftriaxone	3 (37.5%)	1 (12.5%)	4 (50.0%)
Cefazolin	5 (62.5%)	–	3 (37.5%)
Levofloxacin	1 (12.5%)	–	7 (87.5%)
Cefexim	4 (50.0%)	–	4 (50.0%)

**Figure 2:**
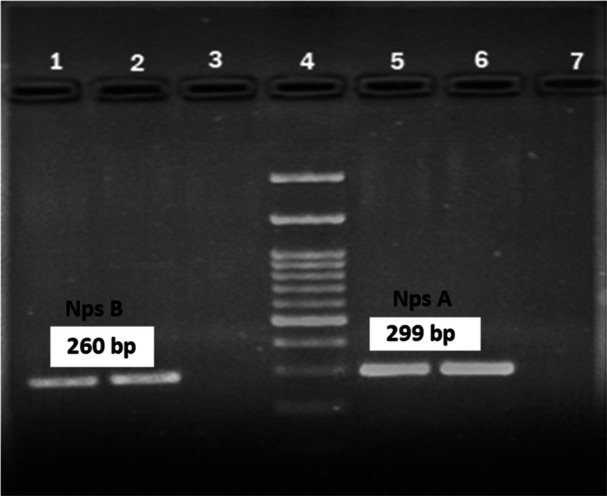
PCR‐based identification of the *npsB* and *npsA* genes. 4: DNA Ladder 100 bp, 1, 2, 5, and 6: Positive sample; 3: Negative sample; 7: Negative control.

The clinical and demographic data of the two participants whose stool sample tested positive for the toxigenic *K. oxytoca* is shown in Table 5. According to the PCR results, out of the 8 *K. oxytoca* strains, 2 (12.5%) were positive for *npsA* and *npsB* genes (Figure 2) and identified as toxigenic *K. oxytoca* strains.

**Table 5:** The clinical and demographic data of two participants whose stool sample tested for toxigenic *K. oxytoca*.

Patients	Sex	Age (years)	Duration of hospitalization	Underlying disease	Diarrhea	Antibiotic usage history	Type of antibiotics (Duration)	Antibiotic resistance profile
1	Female	4	8 days	Wilms tumor	No	Yes	Meropenem (4 days)	Ampicillin, ampicillin‐sulbactam, trimethoprim‐sulfamethoxazole, cefazolin, cefexim
2	Male	9	6 days	Brain tumor	No	No		Ampicillin

## 4 DISCUSSION

Individuals with immunodeficiency and patients with malignancies are susceptible to opportunistic infections.^20^ *K. oxytoca* is a clinically significant opportunistic pathogen and can cause numerous infections in patients with immune deficiency, children, and newborns. Contaminated hospital equipment and healthcare workers can act as a main reservoir for antibiotic resistance *K. oxytoca* isolates.^2^ This study surveyed the frequency of cytotoxin‐producing strains of *K. oxytoca* and their antibiotic susceptibility profile in a cohort of children with different malignancies admitted to Mofid Children's Hospital in Tehran, Iran.

The present study revealed that *K. oxytoca* was found in 2.9% of the stool samples of participants. Our findings are in agreement with those of previously published studies from Spain^9^ and the USA,^21^ which reported that the frequency of *K. oxytoca* among oncology patients was 5.2% and 1.94%, respectively. In contrast, in studies performed by Singh et al. from India^6^ and Carrie et al. from France,^7^ the prevalence of *K. oxytoca* was low. These studies found that the prevalence of *K. oxytoca* in patients was 0.13% and 0.21%, respectively. Results of another study performed by Abbas et al. from Iraq revealed that 67% of patients with colorectal cancer were positive for *K. oxytoca*.^20^ The detected differences in proportions of *K. oxytoca* could be due to differences in the population under investigation, the specimen type, specimen size and applied detecting methods.

The pathogenicity of *K. oxytoca* strains is associated with cytotoxin production ability and most infections are caused by toxigenic strains.^22^ The synthesis of cytotoxin in *K. oxytoca* strains occurs through a bimodular NRPS pathway that has three proteins: NpsA, ThdA, and NpsB. Therefore, in the present study, the prevalence of toxin‐producing *K. oxytoca* strains was identified using specific primers targeting the *npsA* and *npsB* genes. According to the PCR results, 12.5% of *K. oxytoca* isolates were positive for *npsA* and *npsB* genes and identified as toxigenic *K. oxytoca* strains. These results are in line with previously published studies by Schwetz et al. from Austria^23^ and Validi et al. from Iran,^24^ which reported that the incidence of toxigenic *K. oxytoca* among patients was 18.2% and 12%, respectively. However, the obtained results are not consistent with those of previous studies by Greimel et al. in Austria,^12^ Abbas et al. in Iraq,^20^ Hogenauer et al. in Austria,^25^ Joainig et al. in Austria,^26^ and Smith et al. in Canada.^22^ These studies found that 66%, 100%, 100%, 27%, and 23.8% of *K. oxytoca* strains were toxigenic.

Variation in the prevalence of toxigenic *K. oxytoca* among studies can be due to the use of different sample types and types of detection methods.^4^, ^27^ In most cases, *K. oxytoca* strains with toxin‐production ability are found in stool samples. Studies have revealed that *K. oxytoca* strains isolated from other samples such as blood or other infections such as respiratory tract infections cannot produce toxins.^6^, ^12^ In general, it is revealed that positive results of PCR assay cannot confirm the toxicity of isolates. The toxin production ability of *K. oxytoca* strains should be confirmed by culture‐based MTT assay and mass spectrometry‐based metabolomic analysis methods.^11^, ^22^

Results obtained from the antibiotic susceptibility test showed that *K. oxytoca* strains had the highest resistance rates to ampicillin and cefazolin, respectively. Similarly, low susceptibility rates were reported by Alikhani et al., in 2016,^17^ Chakraborty et al., in 2016,^28^ and Rønning et al., in 2019.^29^ The mentioned studies reported a ≥80% resistance of *K. oxytoca* strains to ampicillin.

In contrast, we showed that imipenem, meropenem, ciprofloxacin, amikacin, and levofloxacin were the most effective antibiotics against this bacterium. Similarly, Abdulla et al. from Iraq revealed that most *K. oxytoca* isolates were susceptible to imipenem and meropenem.^30^ Puche et al. from Spain reported that all *K. oxytoca* strains were susceptible to imipenem, meropenem, ceftazidime, cefoxitin, cefepime, and aztreonam.^31^ Moreover, Amaretti et al. from Italy, revealed that all *K. oxytoca* strains were susceptible to the gentamicin, amikacin, ciprofloxacin, trimethoprim‐sulfamethoxazole, amoxicillin‐clavulanic acid, cefotaxime, ceftazidime, and piperacillin‐tazobactam.^15^

However, the results of several other related studies were relatively incompatible with our findings. Singh et al. revealed that 58% of isolates were resistant to imipenem and meropenem.^6^ Vazquez et al. from Spain reported that the susceptibility rates of *K. oxytoca* strains to meropenem, ciprofloxacin, imipenem, and ceftazidime were 36.2%, 18.7%, 11.2%, and 11.25%, respectively.^9^ The emergence of antibiotic‐resistant bacteria and the development of resistance to different classes of antibiotics is a global public health problem.^32^ *K. oxytoca* applies different antibiotic resistance mechanisms such as enzymatic degradation of antibiotics and efficient removal of antibiotics through efflux pumps.^10^

The present study has several limitations including (1) there was no control group and the prevalence of *K. oxytoca* and toxin‐producing isolates was not investigated among healthy people, (2) The current study was a pilot study and due to budget limitations, the toxin production ability was not confirmed using the MTT assay or cell culture method, (3) the sample size was low and there was no assessment of the frequency of virulence genes and the prevalence of genes related to antibiotic resistance.

In conclusion, the current investigation showed that the frequency of *K. oxytoca* in stool samples in Iran is relatively low and that the majority of isolates are sensitive to the tested antibiotics. However, the *K. oxytoca* isolates showed the highest resistance rate to ampicillin, cefazolin, trimethoprim‐sulfamethoxazole, and cefixime. *K. oxytoca* strains is a serious concern in individuals with different malignancies or in immunocompromised patients. Therefore, it is essential to conduct active surveillance of *K. oxytoca* prevalence, develop adequate infection control programs, and utilize appropriate biosafety methods to stop the spread of infections.

## AUTHOR CONTRIBUTIONS

**Nasim Sabzivand**: Investigation; writing—original draft; methodology; formal analysis; data curation. **Shiva Nazari**: Conceptualization; writing—original draft; writing—review and editing; project administration; supervision; data curation; methodology. **Fariba Shirvani**: Conceptualization; writing—original draft; methodology; writing—review and editing; project administration; supervision; investigation. **Leila Azimi**: Investigation; methodology; formal analysis; data curation; writing—original draft; writing—review and editing. **Siavash Salmanzadeh Ahrabi**: Investigation; formal analysis; writing—review and editing; writing—original draft; methodology. **Maedeh Mohammadi Estiri**: Formal analysis; software; methodology; writing—original draft; data curation.

## CONFLICT OF INTEREST STATEMENT

The authors declare no conflict of interest.

## ETHICS STATEMENT

We explained the aims of the present study to children and their parents. A questionnaire was planned for each of the included children and written informed consent was taken from all children and their parents during the study.

## TRANSPARENCY STATEMENT

The lead author Shiva Nazari, Fariba Shirvani affirms that this manuscript is an honest, accurate, and transparent account of the study being reported; that no important aspects of the study have been omitted; and that any discrepancies from the study as planned (and, if relevant, registered) have been explained.

## Data Availability

All authors have read and approved the final version of the manuscript. Dr. Shiva Nazari and Dr. Fariba Shirvani had full access to all of the data in this study and take complete responsibility for the integrity of the data and the accuracy of the data analysis.
